# Mindfulness-Based Treatment for Bipolar Disorder: A Systematic Review of the Literature

**DOI:** 10.5964/ejop.v13i3.1138

**Published:** 2017-08-31

**Authors:** Sanja Bojic, Rodrigo Becerra

**Affiliations:** a School of Psychology & Social Science, Edith Cowan University, Perth, Australia; Webster University Geneva, Geneva, Switzerland; Psychology Department, College of New Rochelle, New Rochelle, NY, USA

**Keywords:** mindfulness, literature review, Bipolar Disorder, Mindfulness Based Cognitive Therapy

## Abstract

Despite the increasing number of studies examining the effects of mindfulness interventions on symptoms associated with Bipolar Disorder (BD), the effectiveness of this type of interventions remains unclear. The aim of the present systematic review was to (i) critically review all available evidence on Mindfulness Based Cognitive Therapy (MBCT) as a form of intervention for BD; (ii) discuss clinical implications of MBCT in treating patients with BD; and (iii) provide a direction for future research. The review presents findings from 13 studies (N = 429) that fulfilled the following selection criteria: (i) included BD patients; (ii) presented results separately for BD patients and control groups (where a control group was available); (iii) implemented MBCT intervention; (iv) were published in English; (v) were published in a peer reviewed journal; and (vi) reported results for adult participants. Although derived from a relatively small number of studies, results from the present review suggest that MBCT is a promising treatment in BD in conjunction with pharmacotherapy. MBCT in BD is associated with improvements in cognitive functioning and emotional regulation, reduction in symptoms of anxiety depression and mania symptoms (when participants had residual manic symptoms prior to MBCT). These, treatment gains were maintained at 12 month follow up when mindfulness was practiced for at least 3 days per week or booster sessions were included. Additionally, the present review outlined some limitations of the current literature on MBCT interventions in BD, including small study sample sizes, lack of active control groups and idiosyncratic modifications to the MBCT intervention across studies. Suggestions for future research included focusing on factors underlying treatment adherence and understanding possible adverse effects of MBCT, which could be of crucial clinical importance.

Psychological interventions implementing mindfulness, such as mindfulness-based cognitive therapy (MBCT; Segal, Williams, & Teasdale, 2002) and mindfulness-based stress reduction (MBSR; Kabat-Zinn, 1990), have gained popularity over the last 15 years. Mindfulness is defined as “paying attention in a particular way: on purpose, in the present moment, and nonjudgementally” (Kabat-Zinn, 1994, p. 4). Mindfulness meditation is postulated to be one of the ‘active ingredients’ of MBCT and involves conscious awareness of the breath or body while noticing thoughts and feelings without judging them or attempting to alter them in any way (Kabat-Zinn, 1990; Segal et al., 2002).

MBCT has been reported to reduce symptoms associated with emotional regulation difficulties in a number of psychological disorders including, major depressive disorder (Kumar, Feldman, & Hayes, 2008) and generalised anxiety disorder (Hofmann, Sawyer, Witt, & Oh, 2010; Kabat-Zinn et al., 1992) and these improvements were maintained over a 3 year follow-up (Miller, Fletcher, & Kabat-Zinn, 1995). Furthermore MBCT significantly reduced the risk of depressive relapse over a period of 12 months in those with three or more prior episodes of major depressive disorder (Ma & Teasdale, 2004; Teasdale et al., 2000).

MBCT has also been associated with decreased rumination of unpleasant emotions (Keng, Smoski, & Robins, 2011) such as worry, anxiety (Chiesa & Serretti, 2009; Jain et al., 2007), feelings of distress (Carlson, Speca, Patel, & Goodey, 2003; Shapiro, Astin, Bishop, & Cordova, 2005), and decreased emotional reactivity (Chiesa, Brambilla, & Serretti, 2010; Chiesa, Calati, & Serretti, 2011) with consistently strong effect sizes (Grossman, Niemann, Schmidt, & Walach, 2004). Furthermore, there is a body of literature supporting MBCT interventions in enhancing self-compassion, positive emotions (Keng et al., 2011), quality of sleep (Brand, Holsboer-Trachsler, Naranjo, & Schmidt, 2012; Carlson & Garland, 2005; Shapiro, Bootzin, Figueredo, Lopez, & Schwartz, 2003), levels of attention, memory, executive functions (Becerra, D’Andrade, & Harms, 2016; Chiesa et al., 2011) and improved emotional regulation abilities (Chiesa et al., 2010; Segal et al., 2002). Additionally, MBCT has been shown to have equivalent results to a course of antidepressant medication maintained over a one-year follow up (Kuyken et al., 2008).

In contrast, a recent comprehensive meta-analysis of 209 studies found that mindfulness interventions were not more effective than traditional CBT and were associated with lower attrition rates (16.25%) compared to CBT interventions (22.5%) (Khoury, Lecomte, Fortin, & Hofmann, 2013). Another review of 24 studies found that almost half of the studies did not find any significant interactions between mindfulness practice and treatment outcome (Vettese, Toneatto, Stea, Nguyen, & Wang, 2009). In addition, it has been reported that mindfulness interventions is unrelated to reduction of symptoms of anxiety and depression (Ramel, Goldin, Carmona, & McQuaid, 2004) or reduction in depression severity (Jermann et al., 2013; van Aalderen et al., 2012; Williams, Teasdale, Segal, & Soulsby, 2000). Others reported that mindfulness interventions did not contribute to improvements in depression, anxiety or impulsivity in patients with Borderline Personality Disorder (Sachse, Keville, & Feigenbaum, 2011) and failed to achieve reduction in worry levels required to meet standard recovery criteria for people with generalised anxiety disorder (Craigie, Rees, Marsh, & Nathan, 2008). These mixed findings about the effectiveness of mindfulness interventions in reducing symptoms associated with various psychological disorders requires further research.

Bipolar Disorder (BD) is a chronic mood disorder characterised by episodes of depression and/or mania (Johnson, 2005; Trede et al., 2005), emotional regulation difficulties (Green, Cahill, & Malhi, 2007; Gruber, Eidelman, & Harvey, 2008) and high comorbidity with anxiety disorders (approximately 62%) (Simon et al., 2005). A number of recent studies have investigated the effectiveness of MBCT on reduction of some of the symptoms associated with BD. Despite several psychological interventions for BD and effective maintenance medication, 50% of BD patients relapse within the first year (Rush et al., 2006) and 70-73% relapse within five years (Gitlin, Swendsen, Heller, & Hammen, 1995; Perry, 1999). Clinical and epidemiological studies have documented that despite pharmacotherapy, patients with BD often continue to experience residual mood symptoms even during the euthymic stage (Judd et al., 2008). The high rate of relapse and reported experienced residual symptoms by many BD patients suggests that there is a gap in current BD treatment. The National Institute of Clinical Excellence (NICE, 2016) recommends MBCT as a relapse prevention approach for patients with a history of depressive episodes, which appears to have prompted studies to investigate MBCT in managing symptoms of BD.

Mixed findings have been reported on the effectiveness of MBCT in BD, in particular when examining the effects of MBCT on depressive symptoms. Although the majority of the studies suggest that MBCT is effective in reducing depressive symptoms associated with BD (Deckersbach et al., 2012, Kenny & Williams, 2007; Miklowitz et al., 2009; Perich, Manicavasagar, Mitchell, & Ball, 2013b; Van Dijk, Jeffrey, & Katz, 2013; Williams et al., 2008), others have reported no difference between participants’ pre and post treatment scores (Ives-Deliperi, Howells, Stein, Meintjes, & Horn, 2013; Perich et al., 2013a). In fact, some studies have reported worsening of depressive symptoms for certain individuals when comparing their pre and post treatment scores (Weber et al., 2010).

Given these inconsistent findings, a critical literature review is needed; specifically examining the effects of MBCT on symptoms associated with BD. Therefore, the aim of the present systematic literature review was to (i) critically review all available evidence on MBCT in BD, (ii) discuss clinical implications of using MBCT in treating patients with BD and to (iii) provide a direction for future research.

## Method

### Data Sources and Search Strategies

A systematic search was conducted through PsychINFO, Medline, PubMed, PsycARTICLES, Google Scholar databases and reference sections of journal articles to obtain relevant literature from the first available date up to and including January 2015.

Key words used in the search were separated into two groups and joined by “AND’ operators. The first group of words identified BD patients; “Bipolar”, “Bipolar Disorder”, “Bipolar Depression”, “Manic”, “Mania”, “Manic Depression”, “Manic Depressive” and “Manic Disorder”. The second group identified Mindfulness; “Mindfulness”, “Mindfulness*”, “Meditation”, “Mindfulness Based Cognitive Therapy”, “Mindfulness Based Stress Reduction”, “Mindfulness Training Meditation” and “Breath Counting”.

### Inclusion and Exclusion Criteria

**Figure 1:**
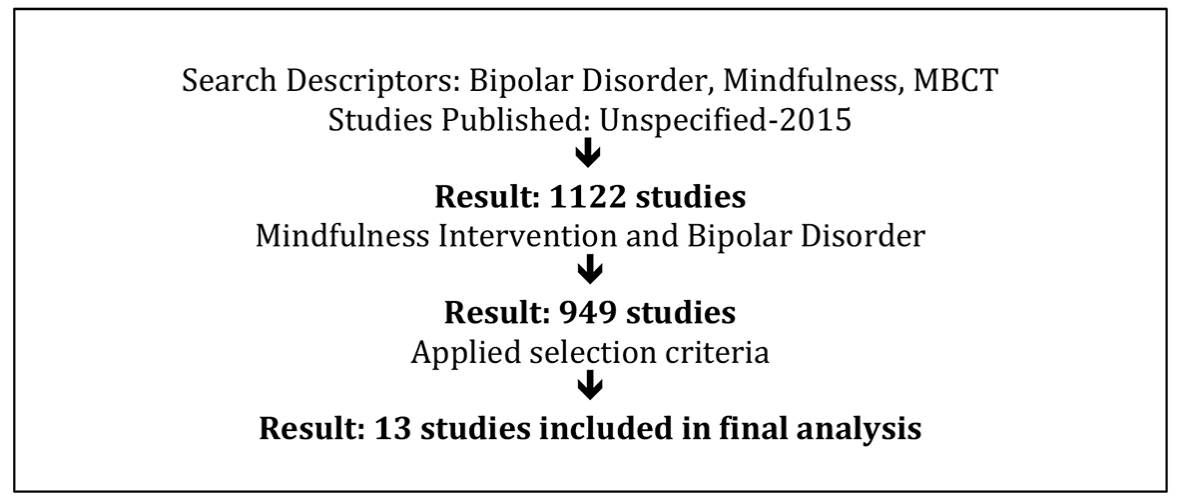
Flow chart of study inclusion/exclusion process.

Studies were included in the review if they met the following criteria: (i) included BD patients (ii) presented results separately for BD patients and the control group (where a control group was available), (iii) implemented MBCT, (iv) were published in English, (v) were published in a peer reviewed journal and (vi) reported results for adult participants. Only original studies were included in the present review, no studies were included that were literature reviews. Studies unrelated to the topic under consideration, studies that involved child participants, duplicate articles across searchers and studies that did not include both BD patients and a mindfulness intervention were excluded. There were 13 studies that met the selection criteria (See Table 1). A flowchart of the study selection process is depicted in Figure 1.

### Data Extraction

The following data were recorded for each study: information about the sample (e.g., the number of participants, diagnosis of BD and comorbidities); information about the intervention (e.g., duration of treatment, type of mindfulness treatment and attrition rates); information about the study (publication year and whether the study was randomized); information about the dependent measures used (e.g., clinical scales that were administered to compare the pre and post scores and to assess mindfulness); and the main findings of each study. Comparison of some of the characteristics (e.g., number of participants, length of intervention, main findings etc.) of the 13 studies included in the present review are presented in Table 1. Studies were grouped into themes such as studies that found that mindfulness intervention was beneficial in reducing symptoms of BD and those that found no differences between pre and post test scores on relevant domains. The areas examined in the present review included effect of mindfulness on major symptoms associated with BD (emotional regulation, depressive, manic/hypomanic and anxiety symptoms); effects of mindfulness on cognitive functioning of BD patients; characteristics of mindfulness intervention in BD (e.g., measuring mindfulness, different changes to the MBCT interventions authors made across studies, attrition rates and adverse effects); limitations of current research and recommendations for future studies.

**Table 1:** Selected Studies Depicting Participants, Groups, Attrition, Clinical Scales, Mindfulness Practice, and Main Outcome

Study	Participants	Control Group	Attrition	Clinical Scales	Mindfulness	Main Outcome
Deckersbach et al. (2012)	*N* = 12 12 euthymic BD patients with moderate residual symptoms BD I (9) = 7 females, 2 males BD II (3) = 2 females, 1 male	No control group Compared pre, post treatment and 3 month follow up	25% (3)	Five-Factor Mindfulness Questionnaire (FFMQ) Hamilton Depression Scale (HAM-D) Young Mania Rating Scale (YMRS) Penn State Worry Questionnaire (PSWQ) Response Style Questionnaire (RSQ) Emotion Reactivity Scale (ERS) Clinical Positive Affective Scale (CPAS) Psychological Well-Being Scale (PWBS) The Longitudinal Interval Follow-up Evaluation –Range of Impaired Functioning Tool (LIFE-RIFT)	12 weekly 2h MBCT sessions	Increased mindfulness, lower residual depressive mood symptoms, less attentional difficulties, and increased emotion-regulation abilities, improved psychological well-being, positive affect, and psychosocial functioning.
Stange at al. (2011)	*N* = 12 12 Euthymic BD patients with moderate residual depressive and varying degrees of residual manic symptoms.	No Control condition	25%	Five-Factor Mindfulness Questionnaire (FFMQ) Hamilton Depression Scale (HAM-D) Young Mania Rating Scale (YMRS) The Frontal Systems Behavior Scale (FrSBe) The Behavior Rating Inventory of Executive Function (BRIEF)	12 weekly 2h MBCT sessions	MBCT showed improvement in executive functioning and memory to levels comparable with normative samples. Improvements in many areas of cognitive functioning, particularly memory and task monitoring, were maintained at the follow-up evaluation 3 months after treatment.
Chadwick et al. (2011)	*N* = 12 12 BD (7 men, 5 women) experiencing mild to moderate depression or elation symptoms.	No control condition	0 dropped out	Semi structured interview	90 min MBCT sessions for 8 weeks plus 6 week booster session. (practiced MBCT for at least 18 weeks)	All participants reported subjective benefits and challenges of mindfulness practice. Seven themes emerged: Focusing on what is present; clearer awareness of mood state/change; acceptance; mindfulness practice in different mood states; reducing/stabilizing negative affect; relating differently to negative thoughts; reducing impact of mood state.
Williams et al. (2008)	*N* = 68 33 Euthymic BD (24 unipolar, 9 bipolar) with a history of serious suicidal ideation/behaviour Follow up data was available for 28 participants in MBCT (21 unipolar and 7 bipolar)	35 in wait-list control condition (27 unipolar, 8 bipolar). Follow up available for 27 in wait list condition (20 unipolar and 7 bipolar)	18% (15) did not attend first assessment 15% (5) did not complete follow up from MBCT group and 23% (8) did not complete follow up from wait list condition.	Mini International Neuropsychiatric Interview (MINI) Beck Depression Inventory (BDI-II) Beck Anxiety Inventory (BAI)	2 hours MBCT sessions for 8 weeks	Improved anxiety (specific to BD group). Both BD and MDD groups in MBCT showed reductions in residual depressive symptoms when compared to those in the waitlist condition.
Howells et al. (2012)	*N* = 21 12 euthymic BD patients (10 females, 2 males)	9 healthy control participants (7 females, 2 males)	Not reported-appears to be 0	Structured Clinical Interview (SCID) Young Mania Rating Scale (YMRS) Hospital Anxiety and Depression Scale (HADS)	8 week MBCT	Brain activity: individuals with BD showed significantly decreased theta band power, increased beta band power, and decreased theta/beta ratios during the resting state, eyes closed, for frontal and cingulate cortices. Post MBCT intervention there was improvement over the right frontal cortex (decreased beta band power) in the BD group. Brain activation: individuals with BD showed a significant P300-like wave form over the frontal cortex during the cue. Post MBCT intervention the P300-like waveform was significantly attenuated over the frontal cortex.
Miklowitz et al. (2009)	*N* = 22 22 euthymic BD (I = 14; BD II = 8) patients (16 females, 6 male). 16 completed MBCT	No control group Compared pre-post measures	27% (6) dropped out	MINI International Neuropsychiatric Interview Hamilton Rating Scale for Depression (HRSD) Young Mania Rating Scale (YMRS) Beck Depression Inventory (BDI-II) Beck Anxiety Inventory (BAI) Beck Scale for Suicide Ideation (BSSI)	2 hour MBCT sessions for 8 weeks	Reductions were observed in depressive symptoms and suicidal ideation and to a lesser extent manic symptoms and anxiety.
Perich et al. (2013a)	*N* = 95 48 BD (I or II)	47 BD- TAU	29% 14 (10 dropped out, 4 did not start MBCT) 22 (18 drop outs and 4 did not start)-47% TAU	Structured Clinical Interview for DSM-IV-TR Disorders (SCID-I) Young Mania Rating Scale (YMRS) Montgomery-Asberg Depression Rating Scale (MADRS) Composite International Diagnostic Interview (CIDI) Depression Anxiety Stress Scale (DASS) State/Trait Anxiety Inventory (STAI) Dysfunctional Attitudes Scale 24 (DAS-24) Response Style Questionnaire (RSQ) Mindful Attention Awareness Scale (MAAS)	2 h sessions for 8 weeks.	There was no significant reduction in time to depressive or hypo/manic relapse, total number of episodes or mood symptom severity at 12 month follow up.
Perich et al. (2013b)	*N* = 34 34 completed MBCT 23 BD (7 male; 16 female) completed homework 22 (8 males; 14 females) completed 12 moth follow up	No control group Compared baseline scores, post treatment and 12 month follow up measures.	29% dropped out of MBCT 68% did not provide information about homework	Young Mania Rating Scale (YMRS) Montgomery-Asberg Depression Rating Scale (MADRS) Composite International Diagnostic Interview (CIDI) Structured Clinical Interview for DSM-IV-TR Disorders (SCID-I) Depression Anxiety Stress Scale DASS, State/Trait Anxiety Inventory (STAI) Mindful Attention Awareness Scale (MAAS) Toronto Mindfulness Scale (TMS)	2 hour MBCT sessions for 8 weeks Follow up testing at 12 months	A greater number of days meditating during the 8 week treatment was related to lower depression scores at 12 month follow up. MBCT was associated with improvements in anxiety and depression if practiced for a minimum of 3 times per week.
Weber et al. (2010)	*N* = 23 23 BD (I, II and NOS) participants 15 BD attended the over 4 MBCT sessions (11 female, 4 male).	No Control group Compared pre and post scores of various clinical scales	35% 8 dropped out (6 dropped out after less than 4 sessions and 2 did not start intervention)	Young Mania Rating Scale (YMRS) Montgomery-Asperg Depression Rating Scale (MADRS) Beck Depression Inventory (BDI-II) The Kentucky Inventory of Mindfulness Skills (KIMS).	2 hour MBCT sessions for 8 weeks plus 2 hours booster session 3 months after 8 week treatment.	There was no significant increase in mindfulness skills following treatment. Mindfulness practice decreased over time. Change in mindfulness skills was significantly associated with change in depressive symptoms between pre and post MBCT.
Ives-Deliperi et al. (2013)	*N* = 33 23 BD with mild to moderate subthreshold symptoms (<14 YMRS and HADS)	7 BD-waitlist 10-healthy controls 16-MBCT	0 drop out	Five-Facet Mindfulness Questionnaire (FFMQ) Symptoms of Stress Inventory (SOSI) Difficulties in Emotion Regulation Scale (DERS) Becks Anxiety Index (BAI) Edinburgh Handedness Inventory (EHI)	8 week MBCT	Following MBCT there were significant improvements in measures of mindfulness, anxiety, emotional regulation, working memory, spatial memory and verbal fluency compared to the waitlist group.
Van Dijk et al. (2013)	*N* = 26 13 BD (I = 5; II = 7)	13 BD (I = 5, II = 7) on waitlist	7.7% 1 dropped out in MBCT group and 1 dropped out of waitlist control	Young Mania Rating Scale The Beck Depression Inventory (BDI-II) The Mindfulness Based Self Efficacy Scale (MSES) The Affective Control Scale (ACS)	90 minute 12 weekly sessions of DBT and Mindfulness	Mindfulness reduced depressive symptoms, improved affective control and improved mindfulness self-efficacy in BD. Mindfulness reduced emergency room visits and mental health related admissions in the 6 months following treatment.
Howells et al. (2014)	*N* = 21 12 Euthymic BD I (10 females, 2 males)	9 healthy controls (7 females, 2 males)	Not reported-appears to be 0 drop out.	Young Mania Rating Scale Hospital Anxiety and Depression Scale Emotional processing was measured by event related potentials (ERP) and heart rate variability (HRV)	8 week MBCT	Following MBCT, BD group showed attenuation of ERP N170 amplitude and reduced HRV HF peak indicating that MBCT may improve emotional processing in BD.
Kenny & Williams, 2007	*N* = 50 50 symptomatic (BDI>19) patients (BD & MDD) 37 female (74%)	No control group Compared pre and post BDI scores.	10% (5) 1 dropped out and 4 did not complete post-treatment measures.	Beck Depression Inventory (BDI-II)	2 hour MBCT sessions for 8 weeks	MBCT improved depression scores with a significant proportion of patients returning to normal/near normal level of mood.

*Note.* BD = Bipolar Disorder; MBCT = Mindfulness Based Cognitive Therapy; MDD = Major Depressive Disorder; TAU = Treatment as usual; *N* = total number of participants.

## Results

Findings were divided into six sections. Each section examined the impact of mindfulness on: emotional regulation, symptoms of depression, symptoms of anxiety, symptoms of mania/hypomania, cognitive functioning and subjective measures of MBCT. Each of the sections was subdivided to include a critical review of research studies that have found support for MBCT and those that have found no significant effects of MBCT on BD.

### Emotional Regulation

The effects of mindfulness intervention on emotional regulation in BD patients were reported in four studies with a total of 92 participants. Emotional regulation is defined as a process people engage in that influences the way people experience (e.g., duration, latency, magnitude and type) and express emotions (Gross, 1998). It consists of physiological and subjective components and thus both aspects have been investigated in the present review. All four studies measured participants’ emotional regulation abilities prior to treatment and after eight weeks of MBCT.

Howells, Rauch, Ives-Deliperi, Horn, and Stein (2014) focused on physiological measures of emotional regulation, such as heart rate variability (HRV). This involved measuring event related potentials (ERP), specifically the ERP N170, and the high frequency (HF) peak of the HRV. The ERP N170 is the most widely used ERP marker of neural processing of faces (Eimer, 2011), whilst the HF peak of HRV serves as a marker of ability to regulate emotions (Telles, Singh, & Balkrishna, 2011), as it is influenced by changes in mood (Hughes & Stoney, 2000).

Howells and colleagues (2014) found that prior to treatment, patients diagnosed with BD (*n* = 12) were found to differ from healthy controls (*n* = 9) on physiological measures of emotional regulation (the effect size was not reported). The BD group had increased ERP N170 amplitude and increased HRV HF peak, suggesting that the BD group had impaired emotional processing prior to treatment when compared to healthy controls (Howells et al., 2014). When participants diagnosed with BD (*n* = 12) engaged in eight weeks of MBCT, there was a decrease in their ERP N170 amplitude and decreased HRV HF peak, indicating improved emotional processing (the effect size was not reported; Howells et al., 2014).

Similar findings were reported by Ives-Deliperi and colleagues (2013), who used the Difficulties in Emotion Regulation Scale (DERS) to measure participants’ emotional regulation abilities. The DERS is a self-reported instrument that measures various aspects of regulating emotions (Gratz & Roemer, 2004). Ives-Deliperi and colleagues (2013) found that BD patients (*n* = 16) reported significantly more difficulties in managing emotions (prior to treatment) when compared to healthy controls (*n* = 10) (the effect size was not reported). This study reported that MBCT intervention was effective in significantly reducing emotion dysregulation scores (the effect size was not reported) for BD patients who prior to treatment had mild or sub threshold symptoms (<14 on Young Mania Rating Scale and Hospital Anxiety and Depression Scale; Ives-Deliperi et al., 2013).

Van-Dijk and colleagues (2013) used the Affective Control Scale (ACS), which is a 42 item self-report questionnaire developed to measure participants’ belief in their ability to control their emotions (Williams, Chambless, & Ahrens, 1997). BD patients were found to struggle with regulating their emotions prior to engaging in MBCT, when emotional regulation was measured using the ACS scale (Van Dijk et al., 2013) (the effect size was not reported). This study found that participants diagnosed with BD who participated in MBCT (*n* = 12), reported increased self-efficacy and belief in having more control over their emotions, when compared to participants diagnosed with BD who were on the waitlist control condition (*n* = 12; the effect size was not reported).

Deckersbach and colleagues (2012) used the Emotion Reactivity Scale (ERS; 21-item) a self-report instrument designed to measure emotional sensitivity, intensity, and persistence (Nock, Wedig, Holmberg, & Hooley, 2008). BD participants who engaged in MBCT reported that they were more aware of internal and external stimuli, were able to respond less judgmentally to their thoughts and feelings and were less reactive to their inner experiences (Deckersbach et al., 2012). There was a medium to large effect size for decreased rumination (Cohen’s *d* = 1.02), worry (Cohen’s *d* = 1.33), reduced attentional difficulties (Cohen’s *d* = 1.50) and increased emotional regulation measured by the ERS (Cohen’s *d* = 0.68) (Deckersbach et al.). It was further noted that there was a linear improvement (from pre-treatment to follow up) in emotional regulation abilities and positive interpersonal relationships, which may suggest that improved abilities to regulate emotions had a positive impact on participants’ interpersonal relationships (Deckersbach et al., 2012).

The current research on MBCT in BD consistently indicates that patients with BD have difficulty regulating their emotions prior to treatment and that engaging in MBCT resulted in improvements in their emotional regulation abilities. These findings were supported by physiological measures (e.g., HRV; Howells et al., 2014) and a number of subjective processes (e.g., ERS, ACS and DERS) (Deckersbach et al., 2012; Ives-Deliperi et al., 2013; Van Dijk et al., 2013). However, it remains unclear whether these treatment gains were maintained long-term, as this was not investigated by any of the current studies.

### Depressive Symptoms

Depressive symptoms were measured using various self-report and clinician rated scales in nine out of the 13 reviewed studies, with a total of 363 participants. The literature in this area reflected mixed findings with six studies reporting that MBCT was effective at reducing depressive symptoms in BD, whilst three studies did not find significant differences in depression scores post-MBCT treatment.

Perich, Manicavasagar, Mitchell, Ball, and Hadzi-Pavlovic (2013a) measured symptoms of depression using the Montgomery-Asberg Depression Rating Scale (MADRS; Montgomery & Asberg, 1979), which is a 10 item clinician administered scale used to assess the severity of depressive symptoms with good internal consistency (Cronbach’s *alpha* = 0.85; Hermens et al., 2006) and inter-rater reliability (0.76; Davidson, Turnbull, Strickland, Miller, & Graves, 1986).

Perich and colleagues (2013a) conducted a randomized controlled trial comparing MBCT (*n* = 22) for BD patients to Treatment As Usual (TAU) (*n* = 12), over a 12-month period. This study found that the interaction of treatment by time for depressive scores was not significant, indicating that depressive scores did not significantly reduce over the 12-month period. This also implied that participating in MBCT did not delay time to reoccurrence of a depression episode when compared to the TAU group. Those who continued to practice meditation throughout the 12- month follow-up period did not report any significant reductions in psychiatric symptomatology compared to those that had not (Perich et al., 2013b).

These findings were supported by Ives-Deliperi and colleagues (2013) who measured symptoms of depression using the Hospital Anxiety and Depression Scale (HADS; Zigmond & Snaith, 1983), which has acceptable (Cronbach’s *alpha* = 0.67) to good (Cronbach’s *alpha* = 0.90) internal consistency (for the depression subscale; Bjelland, Dahl, Haug, & Necklemann, 2002). In this study the authors compared the effects of MBCT (*N* = 33) on depressive symptoms in BD treatment group (*n* = 16), when compared to the BD waitlist control (*n* = 23) and healthy control group (*n* = 10) (Ives-Deliperi et al., 2013). This study found that MBCT did not significantly reduce depression scores, as measured by the HADS scale.

Similar findings were reported in another study (Weber et al., 2010) where depression was measured using the MADRS (this scale was used by Perich et al., 2013b and is described above in more detail) and the Beck Depression Inventory (BDI-II), a well-established self-report questionnaire comprised of 21 items to measure the severity of depression (Beck, Steer, Ball, & Ranieri, 1996). Weber and colleagues (2010) found no significant change between the baseline (*n* = 23), post treatment (8 weeks; *n* = 11) and the 3-month follow up (*n* = 9), on the MADRS and the BDI-II scores. In fact, this study found that four patients experienced an increase in their BDI-II scores at the 3-month follow up. The underlying reasons behind increased depression scores in one scale (BDI-II) measuring depressive symptoms, but not the other (MADRS), were not investigated or reported in this study and therefore remain unclear.

Despite no evidence for reduction in the depressive symptoms in this study (Weber et al., 2010), the majority of participants rated MBCT as helpful in the program evaluation questionnaire. The questionnaire was administered to participants after 8 weeks of MBCT intervention and once again following a one-hour booster session that occurred three months after the eight week MBCT intervention. It was reported that 82% of participants in this study expressed that they benefited from the program. This was explored further, with 55% of participants reporting that MBCT helped them manage unpleasant emotions and 45% found MBCT helped them manage negative thoughts. At the 3-month follow up, 67% of BD patients reported that MBCT improved their quality of life. One major difference between this study and other studies that also failed to find improvement in depressive symptoms in BD (Ives-Deliperi et al., 2013; Perich et al., 2013a), was that Weber and colleagues (2010) included a booster session. The effects of booster sessions in BD would need to be investigated further, as it could potentially be the missing link in helping manage depressive symptoms in BD.

Contrary to the above findings, six studies found that MBCT was beneficial in significantly reducing depression symptoms for patients diagnosed with BD. For example Van Dijk and colleagues (2013) conducted a randomized controlled trial where the BD group (participants with moderate to severe depression; *n* = 12) was compared to a waitlist control condition (*n* = 12). Depression was measured using the BDI-II self-report questionnaire. Following treatment (12 weeks of group MBCT), the majority of participants in treatment were classified as having minimal to mild depression and this reduction in scores was significant (the effect size was not reported).

Similar findings were reported by Kenny and Williams (2007) where depressive symptoms were also measured using the BDI-II scores. In this study BD participants who were currently experiencing depression symptoms (*N* = 50) participated in an 8-week MBCT treatment. The study found that participants’ pre and post BDI-II scores reduced significantly, with large effect size (Cohen’s *d* = 1.04). These findings were further supported by Williams and colleagues’ study (2008) where in a randomized controlled trial a significant reduction in BDI-II scores was reported in the treatment group (euthymic BD patients with a history of suicidal ideation or behaviour; *n* = 28) when comparing the baseline and post treatment scores (the effect size was not reported). There was also a significant reduction in depressive symptoms when comparing the BD treatment group (who participated in eight weeks of group MBCT) and the BD waitlist control group (the effect size was not reported). There was no significant difference between the pre to post BDI scores in the waitlist condition (*n* = 27), suggesting that reduction in depressive symptoms can be attributed to treatment.

Support for MBCT in reducing depressive symptoms in BD was not exclusive to studies that measured depressive symptoms using the BDI-II questionnaire. For example, Deckersbach and colleagues (2012) used the clinician rated, Hamilton Depression Rating Scale (HDRS; Hamilton, 1960), which has high reliability and validity (k = 0.73; Rush, 2000). This study found that participating in MBCT (*n* = 12) reduced depressive symptoms in BD with a strong effect size (Cohen’s *d* = 1.02).

Moreover, Miklowitz and colleagues (2009) measured depression using both the HDRS and BDI-II (*n* = 14). The study found that symptoms of depression and suicidal ideation scores improved, as mean HRSD scores dropped by average of 0.37 SD (*d* = 0.37) and BDI-II scores dropped on average by 5 points (Cohen’s *d* = 0.49). There was also significant improvement on the Beck Scale for Suicide Ideation scores (Cohen’s *d* = 0.51), suggesting MBCT may be beneficial for BD patients with a history of suicidal ideation or behaviours.

Perich and colleagues (2013b) measured depressive symptoms using the MADRS (this scale was also used by Perich et al., 2013a and Weber et al., 2010 and is described in more detail above) and found that for the BD group, the number of days spent practicing meditation (following eight weeks of group MBCT) was significantly inversely correlated with the MADRS scores. This means that BD participants (*n* = 22) that practiced mindfulness for more days had lower MADRS scores at the 12-month follow up (the effect size was not reported). There was some evidence to suggest that mindfulness meditation practice was associated with improvements in depression symptoms if a certain minimum amount (three times a week or more) was practiced weekly throughout the eight-week MBCT program. This suggested that when more time was dedicated to practicing mindfulness it provided protection for depression symptoms over time.

Considering that the same clinical scales were used in both groups of studies, those that have not found support from MBCT in reducing symptoms of depression in BD (Ives-Deliperi et al., 2013; Perich et al., 2013a) and those that have found significant improvement in depressive scores following MBCT (Deckersbach et al., 2012; Kenny & Williams, 2007; Miklowitz et al., 2009; Perich et al., 2013b; Van Dijk et al., 2013; Williams et al., 2008), it appears that the mixed findings could be attributed to within group differences between the studies. For example each group of authors modified MBCT in different way, as there are no evidence-based recommendations for implementing MBCT in BD treatment.

It was also observed that studies that found support for MBCT in reducing symptoms of depression tended to include more hours of mindfulness during treatment. For example some of these studies implemented 12 weeks of two-hour MBCT sessions (Deckersbach et al., 2012; Van Dijk et al., 2013), while those that found no differences in depression scores, implemented only eight weeks (two-hour sessions) of MBCT (Ives-Deliperi et al., 2013; Perich et al., 2013a; Weber et al., 2010). Weber and colleagues (2010) included a booster session, three months after the eight-week intervention and although this study also failed to find significant differences, participants reported they found the intervention beneficial. Therefore, it appears that when it comes to depression symptoms in BD, MBCT seems to be more effective when treatment is held for a minimum of 12 weeks, or when booster sessions are included after 8 weeks.

### Anxiety Symptoms

Anxiety was reported in four out of 13 studies with a total of 157 participants. Unlike the findings for depressive symptoms, the literature reflected more robust effects of MBCT in studies that measured changes in anxiety symptoms. Studies consistently reported that MBCT was helpful in either significantly reducing or preventing anxiety symptoms from increasing over time.

Ives-Deliperi and colleagues (2013) measured anxiety using the Beck Anxiety Inventory (BAI; Beck & Steer, 1990), which has shown good reliability (Cronbach’s *alpha* = 0.82; Contreras, Fernandez, Malcarne, Ingram, & Vaccarino, 2004) and the Hospital Anxiety and Depression Scale (HADS; Zigmond & Snaith, 1983), which has adequate to good (Cronbach’s *alpha* = 0.68 to 0.93) internal consistency (Bjelland et al., 2002). This study found that prior to treatment BD participants (*n* = 16) reported significantly increased levels of anxiety when compared to the healthy controls (*n* = 10; Ives-Deliperi et al., 2013). Eight weeks of group MBCT resulted in significant reductions in anxiety in the BD treatment group (*n* = 16; the effect size was not reported), but not in the BD waitlist group (*n* = 7), indicating that decrease in anxiety could be attributed to treatment. These findings were consistent with another study, which also found modest improvements in the pre to post intervention BAI scores of BD patients (Cohen’s *d* = 0.23), following eight weeks of group MBCT (Miklowitz et al., 2009).

Williams and colleagues (2008) compared pre and post BAI scores of BD patients in remission (with history of suicidal ideation/behaviour; *n* = 28) and a waitlist control group (*n* = 27). The study found that at baseline there were no differences between the BAI scores of BD treatment and BD waitlist group. Following eight weeks of group MBCT, those who participated in the treatment condition had significantly lower BAI scores compared to the waitlist group (the effect size was not reported). However, those that participated in treatment had no significant change in BAI scores when comparing their pre and post treatment scores, whilst those on the waitlist had significant increase in BAI scores over time (the effect size was not reported). In other words, although MBCT did not decrease BAI scores following treatment, participating in MBCT prevented anxiety from increasing over time. The major depression group showed no differences in BAI scores, suggesting that protective effect of MBCT on levels on anxiety was specific to the BD group.

Perich and colleagues (2013b) measured anxiety symptoms using a self-report Depression and Anxiety Stress Scale (DASS; Lovibond & Lovibond, 1995), which has adequate reliability for measuring symptoms of depression (Cronbach’s *alpha* = 0.95; Crawford & Henry, 2003) and State/Trait Anxiety Inventory (STAI) scale (Spielberger, 1983), with good internal consistency for the state (ranging from Cronbach’s *alpha* = 0.90 to 0.91) and trait scales (ranging from Cronbach’s *alpha* 0.86 to 0.85, Spielberger, 1983).

Although the study (*N* = 34) found that anxiety scores did not improve over the 12 month follow up period following engagement in eight week MBCT group intervention, BD participants had improvement in anxiety symptoms (STAI trait anxiety scores; Perich et al., 2013b). The improvements were noted when mindfulness was practiced once a day for at least three days a week during the eight-week treatment period (the effect size was not reported). This positive effect of MBCT on levels of anxiety was consistent with studies investigating MBCT in non-clinical populations (Schenström, Rönnberg, & Bodlund, 2006).

MBCT has shown promising positive effects on managing symptoms of anxiety in patients with BD. Three studies have reported that engaging in eight weeks of MBCT resulted in significant decreases in anxiety scores (Ives-Deliperi et al., 2013; Miklowitz et al., 2009; Williams et al., 2008). One study had also reported that engaging in MBCT was protective in the sense that it stopped anxiety scores increasing over time (Williams et al., 2008). Most importantly MBCT was effective at reducing anxiety scores when mindfulness was practiced for a minimum of three days per week (Perich et al., 2013b).

### Mania/Hypomania Symptoms

Six out of the 13 studies measured mania/ hypomania symptoms before and after MBCT treatment (*N* = 209). Other studies measured symptoms of mania prior to treatment, as an exclusion criterion for participating in the MBCT intervention. All of the six studies measured symptoms of mania using the Young Mania Rating Scale (YMRS), which is a clinician-administered scale with good inter-rater reliability (Young et al., 1978). Out of the six studies that measured symptoms of mania post-MBCT treatment, five found no significant differences in the YMRS scores after participating in MBCT (Deckersbach et al., 2012; Howells et al., 2014; Perich et al., 2013a; Perich et al., 2013b; Weber et al., 2010). Only one study (Miklowitz et al., 2009) reported significant reduction in the manic symptoms following participation in MBCT.

Deckersbach and colleagues (2012) (*N* = 12) reported that participants in their study had no to low residual manic symptoms prior to participating in MBCT. After treatment there was no significant difference in the YMRS scores. It was noted that there was an increase in YMRS for one participant, which appeared to be caused by elevation in his mood. Similarly, Weber and colleagues (2010) (*N* = 23) only included people with BD who were in remission and scored less than eight points on the YMRS. The study found no significant differences in the YMRS scores after participating in MBCT, when comparing the baseline scores with the one month follow up and three month follow up scores.

Further to this, Perich and colleagues (2013b) found that the number of days participants dedicated to engaging in mindfulness activities was not significantly correlated with YMRS scores post-treatment (*N* = 34). In addition, Perich and colleagues (2013a) found no significant differences between the pre and post YMRS scores (*N* = 95), as well as no significant differences between the YMRS scores between the treatment group and the TAU group following MBCT treatment. This study concluded that MBCT had no impact on reducing the risk of reoccurrence of hypo/manic symptoms, total number of episodes or mood symptom severity at the 12 months follow up. It is of note that all BD participants were euthymic and complying with mood stabilising medication for the duration of the studies.

Howells and colleagues (2014) also failed to detect significant differences in the pre and post treatment YMRS scores (*N* = 21), however they reported that although participants were already euthymic at the baseline measure, their YMRS scores reduced slightly after engaging in MBCT (Cohen’s *d* = 0.17). Unlike other studies, Miklowitz and colleagues (2009) included participants with subsyndromal mania and hypomania (*N* = 22). Contrary to the above studies, this study found significant reductions in the YMRS scores when they compared the pre and post MBCT scores (Cohen’s *d* = 0.17).

In summary, five studies have reported that participants with BD experienced no significant differences in symptoms of mania/hypomania following engagement in MBCT (Deckersbach et al., 2012; Howells et al., 2014; Perich et al., 2013a; Perich et al., 2013b; Weber et al., 2010). One of these studies reported a slight reduction in YMRS scores, although it was not significant (Howells et al., 2014). One study found that participation in MBCT significantly reduced symptoms of mania (Miklowitz et al., 2009). It appears that when people with BD are euthymic and complying with their mood stabilising medication, practicing mindfulness does not provide reduction of mania/hypomania symptoms or protection against future episodes. However, when people with BD are experiencing subsyndromal mania, it appears that practicing mindfulness helps reduce the severity of symptoms. Considering that these findings were only reported in one study, they must be interpreted with some caution, and the effects of MBCT on symptoms of mania need to be investigated further.

### Cognitive Functioning

Three studies investigated the effects of MBCT on cognitive functioning of BD patients with a total of 66 participants. All three studies reported that BD patients exhibited deficits in various aspects of their cognitive functioning and that participating in MBCT resulted in improvements in their executive functioning (Ives-Deliperi et al., 2013; Stange et al., 2011), attentional readiness (Howells, Ives-Deliperi, Horn, & Stein, 2012) and memory, which were maintained at a three month follow up (Stange et al., 2011).

Ives-Deliperi and colleagues (2013) compared healthy controls (*n* = 10) with a waitlist BD group (*n* = 7) and BD participants who took part in an eight-week MBCT condition (*n* = 16). The study found that prior to the treatment BD participants scored lower on measures of executive functioning including working memory and inhibition. Significant Blood Oxygenation Level Dependent (BOLD) signal decreases were noted in the medial prefrontal cortex in the BD compared to the control group. This indicated that the BD group had difficulty focusing on the tasks performed. Following MBCT, the study found that the BD treatment group improved in neuropsychological tasks measuring working memory (digit span backward), spatial memory (Rey Complex Figure Recall) and verbal fluency (Controlled Oral Word Association Test).

MBCT resulted in significant improvements in executive performance in the BD treatment group, but not in the BD waitlist group (the effect size was not reported; Ives-Deliperi et al., 2013). Significant BOLD signal increases were observed in the medial prefrontal cortex and posterior cingulate cortex in the BD treatment group, compared to the BD waitlist group, during the mindfulness task (the effect size was not reported). These changes in BOLD signal resulted in an activation pattern more closely resembling those of healthy controls.

Howells and colleagues (2012) found that prior to engaging in MBCT, brain activity for the BD group (*n* = 12) was greater over the frontal cortex and cingulate cortex and showed activation of non-relevant information processing, when compared to the healthy control (*n* = 9) group. This suggested decreased attentional readiness prior to treatment, as theta activity was decreased, beta activity was increased and theta/beta ratios were decreased over the frontal and cingulate cortices. This study found that BD participants had deficits in resting brain activity, which may have reduced their ability to attend to relevant information. After participating in eight week MBCT, the BD group showed changes in the right frontal EEG activity (beta activity was decreased and theta and theta/beta ratios increased), which was associated with slight improvement in attention readiness (the effect size was not reported).

Stange and colleagues (2011) compared pre and post scores on two self-reported scales for measuring cognitive functioning (*N* = 12): the Frontal Systems Behavior Scale (FrSBe; Stout, Ready, Grace, Malloy, & Paulsen, 2003) and the Behavior Rating Inventory of Executive Functioning (BRIEF; Roth, Isquith, & Gioia, 2005). Prior to MBCT treatment, cognitive functioning of BD patients (*n* = 8) was substantially lower than normative comparison samples. The greatest deficits were in the BRIEF metacognition summary scores (comprised of initiate, working memory, plan/organize, task monitor and organisation of materials). After participating in MBCT (12, two-hour group sessions) there were improvements in executive functioning and memory to levels comparable with normative sample with large effects sizes (Cohen’s *d* = 1.13 to 1.39) between pre treatment and post treatment scores on most subscales. Effects of the treatment in terms of improvements in executive functioning, memory and task monitoring were maintained at a three month follow up.

Taken together, results consistently indicate that MBCT is effective in improving cognitive functioning in BD.

### Subjective Measures of MBCT

Three studies (*N* = 36) reported the effect of MBCT on symptoms of BD using subjective measures, such as interviews, case studies and self-rating questionnaires about participants’ experience in taking part in a mindfulness intervention.

Chadwick, Kaur, Swelam, Ross, and Ellett (2011) conducted semi-structured interviews (*N* = 12) and analysed the data using thematic analysis. This study found that MBCT helped participants with BD increase their ability to focus on the present moment, increase their self-awareness and acceptance and improve their ability to manage mood states and negative thoughts. The majority of participants reported that they had to adapt their mindfulness practice to different mood states, as mindfulness practice was more difficult when participants felt depressed (low mood) than when their mood was high (although no one reported experiencing mania). It appeared that having a choice of various mindfulness exercises allowed participants to be responsive to their moods and continue completing their home practice. Participants reported that mindful movement exercise (such as walking mindfully or engaging in gentle yoga exercises) with short mindfulness exercises was particularly helpful while mindfulness of breath (focusing on one’s breathing while seated) was more helpful when mood was high. This information suggests that effective MBCT interventions will need to consider the patient’s mood and energy levels to increase compliance with homework and appropriate level of engagement with treatment.

Weber and colleagues (2010) administered questionnaires to evaluate participants’ experience of eight weeks of MBCT (*N* = 23). The study found that MBCT was well received among participants with BD, with 82% reporting they moderately to very much benefited from MBCT. One month post-MBCT, 55% of participants with BD reported that MBCT helped them to cope with emotions and 45% said it helped them implement more structure in their life and cope with negative thoughts. Three months post-MBCT, 67% reported that MBCT helped them regulate their emotions and 67% reported it improved their overall quality of life. Participants reported that mindful breathing, body scan, sitting meditation and mindful movement exercises were the most beneficial in managing symptoms associated with BD.

Miklowitz and colleagues (2009) included a case vignette of a 36-year-old woman, Sarah, who was diagnosed with BD I. Sarah reported that attending MBCT group helped her “normalise” her experience of living with symptoms of BD. She reported that MBCT helped her slow down her thoughts and take on more of an observer’s role instead of reacting to them. Further to this Sarah reported that MBCT helped her identify her feelings and mood changes earlier. For example, she particularly benefited from the three-minute breathing space exercise to manage her feelings of anger and avoid angry outbursts, which she stated had a positive impact on her intimate relationships.

## Discussion

The present paper aimed to systematically review current literature on effectiveness of MBCT on managing symptoms associated with BD. We reported findings from 13 studies (*N* = 429), which were categorised into six sections, namely, emotional regulation, depression, anxiety, mania, cognitive functioning and subjective measures of MBCT in BD.

MBCT has shown promising positive effects on managing symptoms of anxiety in patients with BD (Ives-Deliperi et al., 2013; Miklowitz et al., 2009; Williams et al., 2008) and preventing anxiety scores from increasing over time (Williams et al., 2008). MBCT was also associated with improved physiological health (e.g., HRV; Howells et al., 2014) and improvements in a number of subjective measures (e.g., ERS, ACS and DERS) of emotional regulation in BD (Deckersbach et al., 2012; Ives-Deliperi et al., 2013; Van Dijk et al., 2013). Furthermore participating in MBCT resulted in improvements in executive functioning (Ives-Deliperi et al., 2013; Stange et al., 2011), attentional readiness (Howells et al., 2012) and memory, with treatment gains maintained at a three month follow up (Stange et al., 2011).

The current literature suggests that MBCT does not provide reduction of mania/hypomania symptoms or protection against future episodes (Deckersbach et al., 2012; Howells et al., 2014; Perich et al., 2013a; Perich et al., 2013b; Weber et al., 2010) in euthymic BD participants. However significant reductions in mania symptoms were reported when participants with residual manic symptoms participated in MBCT (Miklowitz et al., 2009).

The mixed findings between studies that reported no significant differences in symptoms of depression (Ives-Deliperi et al., 2013; Perich et al., 2013a) and those that have found significant improvement in depressive symptoms following MBCT, (Deckersbach et al., 2012; Kenny & Williams, 2007; Miklowitz et al., 2009; Perich et al., 2013b; Van Dijk et al., 2013; Williams et al., 2008) appear to be related to specific modifications of mindfulness interventions across groups. It was found that MBCT was effective at managing symptoms of BD when mindfulness was practiced for a minimum of three days per week (Perich et al., 2013b) or when booster sessions were included (Chadwick et al., 2011; Weber et al., 2010).

Some common limitations were observed in the literature including small sample size, lack of control group, various modifications to MBCT intervention, noncompliance or non reporting of homework completion and a lack of adequate monitoring and reporting of any adverse effects and factors contributing to attrition rates.

### Sample Size and Control Conditions

Most of the studies reviewed in this paper had a small sample size, (ranging from *n* = 8 [Stange et al., 2011] to *n* = 68 [Williams et al., 2008]), consisting of predominantly female participants (72%), as many were ‘pilot’ studies in this area. Some studies did not have an active control group (Chadwick et al., 2011; Deckersbach et al., 2012; Miklowitz et al., 2009; Stange et al., 2011; Weber et al., 2010), which only allowed comparison of pre and post treatment scores. Others included a waitlist condition (Perich et al., 2013a; Van Dijk et al., 2013; Williams et al., 2008), while more recent studies included a healthy control group as a comparison (Howells et al., 2014; Ives-Deliperi et al., 2013).

### Modification of Mindfulness Interventions

One of the limitations of current literature on MBCT in BD is that mindfulness intervention was modified in some way in almost every study, which made it difficult to attribute treatment gains exclusively to MBCT. Considering that there are currently no published guidelines about how to implement mindfulness – based treatment for BD patients, it is understandable that researchers implemented changes to make MBCT more applicable.

It was observed that all 13 studies included in the current review based their MBCT intervention on the program described by Segal and colleagues (2002) and introduced various changes to make treatment more responsive to BD patients. Some studies did not provide information about specific session outlines of their mindfulness treatment (e.g. Howells et al., 2012; Ives-Deliperi et al., 2013; Stange et al., 2011) while others provided a detailed account of their session plans (e.g. Perich et al., 2013a). The inconsistent reporting of the characteristics of mindfulness interventions only allowed for a superficial comparison across studies, as information was limited.

Most of the studies in this review delivered the mindfulness intervention during a weekly two-hour group session for eight weeks. However, some studies (e.g., Weber et al., 2010) reported that they included a two-hour booster session three months after treatment. Another study (Chadwick et al., 2011) delivered MBCT via 90-minute sessions for eight weeks, plus six-week booster sessions, which resulted in participants practicing in MBCT for a minimum of 18 weeks. Other exceptions to the eight week MBCT intervention were reported in Deckersbach and colleagues’ (2012) study where mindfulness was administered in 12 weekly, two-hour sessions, whilst Van Dijk and colleagues (2013) conducted 12, 90-minute sessions.

Some differences were observed in duration and types of mindfulness activities that were practiced during sessions. Williams and colleagues (2008) reported that participants engaged in two-hour meditation practice during the last three weeks of intervention. Other studies had brief mindfulness activities (e.g. body scan, mindfulness of sounds, mindfulness of feelings and mindful walking) of approximately 20 to 30 minutes (Chadwick et al., 2011). Most studies reported approximately 40 minutes of mindfulness meditation practiced during sessions (Perich et al., 2013a) and most introduced mindfulness movement exercises to address attention difficulties (Deckersbach et al., 2012).

All of the reviewed MBCT studies introduced some level of psychoeducation about BD. For example, Weber and colleagues (2010) reported presenting information about mania and hypomania. Similarly, Perich and colleagues (2013a) reported introducing relapse prevention information about BD, depression, hypo/mania and anxiety. Van Dijk and colleagues (2013) extended the psychoeducation part of their intervention to include a session about medication and importance of self-care (e.g., sleep hygiene, eating healthy and abstaining from drugs and alcohol). Deckersbach and colleagues (2012) incorporated daily mood monitoring, emergency plans if mood deteriorated and problem solving focused on reducing the likelihood of dropping out of treatment. As there are currently no specific guidelines for implementing MBCT to manage symptoms of BD, each study included information in the psychoeducation part of their intervention as they saw fit, without providing any empirical evidence to support introducing that particular information. Considering that there is evidence in the literature that psychoeducation alone is effective in managing symptoms of BD (Colom et al., 2009; Stafford & Colom, 2013), this makes attribution of change to MBCT more difficult.

### Homework Completion

It appeared that many participants struggled to complete mindfulness homework, as significant data were lost due to participants forgetting to complete or keep a record of exercises. For example, Perich and colleagues (2013a) reported that only 67% of participants that completed the MBCT program provided information of their homework completion, which resulted in 33% of participants being excluded from the final analysis.

The homework expected from participants also differed in duration, content and frequency across studies. It was observed that some studies required participants to complete a daily 10-minute mindfulness of breath exercise (focusing on one’s breathing; Chadwick et al., 2011), while others required a minimum of 45-minute meditation, six days a week (Williams et al., 2008). Deckersbach and colleagues (2012) indicated that participants in their study completed 40-minute yoga exercises at home, while in another study participants’ homework involved 60 minutes of yoga exercises (Kenny & Williams, 2007). Some studies provided their participants with a CD to help with required homework practice (Weber et al., 2010) while others omitted the CD (Perich et al., 2013b; Weber et al., 2010). For example, Perich and colleagues (2013a) stated that purchase of the book ‘Full Catastrophe Living’ (Kabat-Zinn, 1990) was optional, the yoga CD was omitted and the DVD ‘Healing from Within’ was unavailable for purchase at commencement of the study. Weber and colleagues (2010) reported that instead of providing above-mentioned CDs/DVDs, they recorded their own CD, which was given to participants to help with homework exercises.

These inconsistences in the duration, type, and frequency of mindfulness homework made it difficult to compare across studies and attribute any treatment gains exclusively to MBCT interventions. The high rate of noncompliance with mindfulness home practice also indicates that future research needs to investigate effective strategies to motivate homework completion.

### Attrition Rates

Reasons behind why participants decided not to engage in treatment or to stop attending MBCT before completing the treatment were not explored in any of the studies included in the current review. The average dropout rate in reviewed studies was 16%, which indicated that 84% of participants adhered to treatment. This was comparable to other studies that investigated attrition rates in MBCT interventions in participants with recurrent depression (Crane & Williams, 2010; Kuyken et al., 2008).

There was a trend for those allocated to a wait list condition to have higher dropouts and non-completion than those who participated in MBCT (Perich et al., 2013a). Participants that dropped out from MBCT tended to be younger than those that successfully completed treatment (Miklowitz et al., 2009) and participants that reported a greater number of prior episodes engaged in fewer days of meditation. This appeared to reflect that this group of participants experienced greater difficulty in engaging in mindfulness.

Crane and Williams (2010) investigated attrition rates in mindfulness treatment and found that those that dropped out were significantly younger, less likely to be taking antidepressants, had higher levels of depressive rumination and showed greater levels of problem solving deterioration, than those that completed treatment. This study suggested that participants with high depressive rumination and cognitive reactivity find it particularly difficult to engage in MBCT (Crane & Williams, 2010). Also when dropouts occurred they tended to happen early in treatment (before treatment has started or after attending only one session). Another study (Kuyken et al., 2008) found that some of the common reasons for dropout included disliking the group format and the time commitment involved in mindfulness based treatment. Attrition rates in studies examining effects of MBCT on symptoms associated with BD need to be investigated in future studies.

### Adverse Effects

Potential adverse effects were not adequately investigated or reported in studies that examined effects of MBCT in BD. One study (Deckersbach et al., 2012) reported that there was a small increase in mood elevation at follow up, which was driven by one participant who experienced hypomania. In another study (Miklowitz et al., 2009), one patient had worsening of mania symptoms, however it was noted that overall improvement with depression did not coincide with worsening of mania. Kenny and Williams (2007) reported that four participants had increased BDI (depression) scores. Adverse effects appeared to be rare in MBCT for BD, however it is possible that the numbers of patients that suffered from adverse effects were higher than reported and could include some of the participants that dropped out of treatment.

It was noted that adverse effects were not monitored or recorded routinely in mindfulness interventions in general (Dobkin, Irving, & Amar, 2012). There are documented cases of participants experiencing mania following participating in meditation (Yorston, 2001) and more extreme cases of participants experiencing psychosis following intensive meditation training (Chan-Ob & Boonyanaruthee, 1999; VanderKooi, 1997). For some patients with a history of psychosis it can be beneficial to engage in mindfulness during remission (Bach & Hayes, 2002; Gaudiano & Herbert, 2006), while for others it could aggravate their symptoms (Dobkin et al., 2012). Thus the potential adverse effects of MBCT in BD require further research.

### Conclusion and Recommendations

Mindfulness research in BD is in the early stages and definite conclusions about effectiveness cannot yet be drawn. However, the current review of the literature indicated that MBCT was associated with improvements in emotional regulation (Howells et al., 2014) and reductions in symptom of anxiety (Ives-Deliperi et al., 2013) and depression in BD (Kenny & Williams, 2007). Furthermore, MBCT intervention was associated with improvements comparable to normative samples in several aspects of cognitive functioning (Howells et al., 2014; Stange et al., 2011). These gains were maintained at a three-month follow up (Stange et al., 2011) and some were also maintained at a 12-month follow up (Perich et al., 2013b), however a booster session appeared to be necessary (Deckersbach et al., 2012). Practicing mindfulness for a minimum of three days a week was associated with improvement of depression and anxiety symptoms, and this was recommended as the minimal effective dose of MBCT for BD (Perich et al., 2013b).

MBCT in euthymic BD patients resulted in no significant difference between pre and post mania scores (Deckersbach et al., 2012; Howells et al., 2014), however when participants reported residual mania or hypomania symptoms (prior to MBCT intervention), significant reduction in their mania symptoms was documented, indicating that MBCT may be effective for those patients (Miklowitz et al., 2009). Further studies are needed as symptoms of mania were not adequately examined in the available literature and were in fact considered an exclusion criterium for some studies.

Some of the current studies have not adequately reported and investigated attrition rates or the adverse effects of MBCT. Other limitations of reviewed studies included using various clinical scales to measure mindfulness and modifying mindfulness interventions to varying degrees, which made comparisons across studies difficult and raising serious doubts about treatment integrity. It is recommended for future studies in this area, to focus on implementing a control group, larger sample size, and developing a standard MBCT intervention specifically for BD patients. In addition, future studies should explore potential detrimental effects of MBCT for some people with BD. Overall it can be concluded that MBCT is a promising treatment for BD in conjunction with pharmacotherapy, however further studies are required to investigate long-term effects.
